# *Post-mortem* magnetic resonance microscopy (MRM) of the murine brain at 7 Tesla results in a gain of resolution as compared to *in vivo* MRM

**DOI:** 10.3389/fnana.2014.00047

**Published:** 2014-06-13

**Authors:** Oliver von Bohlen und Halbach, Martin Lotze, Jörg P. Pfannmöller

**Affiliations:** ^1^Institut für Anatomie und Zellbiologie, Universitätsmedizin GreifswaldGreifswald, Germany; ^2^Functional Imaging Unit, Center for Diagnostic Radiology, University of GreifswaldGermany

**Keywords:** spatial resolution, animal scanner, *in vivo*, MRI, segmentation, *post-mortem*, signal-to-noise ratio, contrast-to-noise ratio

## Abstract

Small-animal MRI with high field strength allows imaging of the living animal. However, spatial resolution in *in vivo* brain imaging is limited by the scanning time. Measurements of fixated mouse brains allow longer measurement time, but fixation procedures are time consuming, since the process of fixation may take several weeks. We here present a quick and simple *post-mortem* approach without fixation that allows high-resolution MRI even at 7 Tesla (T2-weighted MRI). This method was compared to *in vivo* scans with optimized spatial resolution for the investigation of anesthetized mice (T1-weighted MRI) as well as to *ex situ* scans of fixed brains (T1- and T2-weighted scans) by using standard MRI-sequences, along with anatomic descriptions of areas observable in the MRI, analysis of tissue shrinkage and post-processing procedures (intensity inhomogeneity correction, PCNN3D brain extract, SPMMouse segmentation, and volumetric measurement). *Post-mortem* imaging quality was sufficient to determine small brain substructures on the morphological level, provided fast possibilities for volumetric acquisition and for automatized processing without manual correction. Moreover, since no fixation was used, tissue shrinkage due to fixation does not occur as it is, e.g., the case by using *ex vivo* brains that have been kept in fixatives for several days. Thus, the introduced method is well suited for comparative investigations, since it allows determining small structural alterations in the murine brain at a reasonable high resolution even by MRI performed at 7 Tesla.

## INTRODUCTION

The murine brain is about 3500 times lighter than the human brain and the small brain size challenges high field imaging with high-quality and high spatial resolution. Absolute scan time with a sufficient signal-to-noise ratio (SNR) and contrast-to-noise ratio (CNR) is dependent on spatial resolution (Buxton, 2009).

Although it is possible to anesthetize mice with isoflurane for 6.5 h without any mortality (Szczesny et al., 2004), practically 120 min is the limit for MRI-measurement duration.

However, these investigations are accompanied by artifacts due to due to circulation and breathing movements (Johnson et al., 1993; Benveniste and Blackband, 2002). For *ex vivo* investigation the use of formaldehyde perfusion-fixed brains has offered the opportunity to use even longer scan times, which resulted in a gain of resolution (Benveniste et al., 2000). These procedures, however, result in quality loss, due to denaturation and cross-linking of proteins (Benveniste and Blackband, 2002). Therefore, staining techniques have been developed such as the most commonly used contrast-enhancing agents (Huang et al., 2009; Kim et al., 2009; Cleary et al., 2011). These techniques are well suited to obtain high-quality scans useful, e.g., for creating atlases of the rodent brain (Ma et al., 2005). However, the use of contrast-enhancing agents on *in situ* brains is very time consuming (Huang et al., 2009; Cleary et al., 2011). Due to this limitation, this technique might not be well suited for performing comparative studies, as, e.g., for analyzing brains of genetically altered animals, in comparison to wild-type littermates within a small time frame. Moreover, a high prevalence of fixation artifacts (approximately in 30% of the samples) has been observed in fixed mouse brain magnetic resonance images (Cahill et al., 2012). This may compromise their use for quantitative morphometric analyses, where accurate anatomical volumes and morphology are essential (Cleary et al., 2011).

To get insight in the usefulness of the introduced *post-mortem* method, we compared this method with *in vivo* and other *ex situ* imaging techniques. For each of these approaches we optimized 7 Tesla MRI scanning procedures. We used three quality criteria: (1) visual differentiation of brain substructures by using a mouse atlas as a reference (Paxinos and Franklin, 2001), (2) brain volume estimation for detecting possible shrinkage in comparison to the *in vivo* situation, (3) the capability for automatized brain segmentation extraction algorithms (SPMMouse; Sawiak et al., 2009). Low capability needed more preprocessing interventions such as intensity inhomogeneity correction (Sled et al., 1998) or brain extraction algorithms (Chou et al., 2011). For the gray and white matter, an averaged SNR was calculated.

## MATERIALS AND METHODS

Scans were performed in a 7 Tesla ClinScan 70/30 animal scanner (Bruker, BioSpin, Ettlingen, Germany). We use a 2 × 2 channel mouse brain coil. Female and male adult C57 wild-type mice were examined. In order to compare the introduced fixation-free *post-mortem* method with *in vivo* and *ex situ* MRI scans, animals were first analyzed using *in vivo* MRI-scanning [see MRI-Scanning (*In Vivo*) section]. Thereafter, mice were euthanized with an overdose of ether (von Bohlen und Halbach et al., 2008), since it does not interfere with neuronal activity as, e.g., isoflurane (Zschenderlein et al., 2011). Thereafter, they were analyzed using *post-mortem* MRI-scanning [see MRI-Scanning (*Post-Mortem*) section].

All animal experiments were performed in accordance with German animal rights regulations and with permission of the Landesamt für Landwirtschaft, Lebensmittelsicherheit und Fischerei (*LALLF*) Mecklenburg-Vorpommern, Germany

Next, the brains were removed and fixed using 4% paraformaldehyde. *Ex situ* MRI scans [see MRI-Scanning (*Ex Situ*) section] were performed at two different time points: (i) some hours after the *post-mortem* scan and (ii) 7 days subsequent to the first *ex situ* scan. All MRI scans were performed at room temperature. All experimental procedures were performed according to permission obtained from local state authorities.

### MRI-SCANNING

#### MRI-scanning (in vivo)

Mice were anesthetized with a mixture of 2.5% isoflurane and oxygen for induction of anesthesia and then transferred to the MRI scanner. A 3D T1-weighted turbo-flash (TFL) sequence [30 transversal slices, 280 μm thickness, no gap, matrix of 512 × 512 pixel, field of view (FoV) = 30 mm, spatial resolution 59 μm × 59 μm × 280 μm, voxel volume 0.97 nl, repetition time (TR) = 2200 ms, echo time (TE) = 4.63 ms, inversion time (TI) = 1000 ms] was used for their examination. The total scan time of the *in vivo* sequence was 1:53 h. During the whole-brain scan respiration rate was monitored with an MR-compatible Small Animal Monitoring and Gating System Respiration Module (SA Instruments, Inc., Stony Brook, NY, USA) and anesthesia with isoflurane (1–2%) and oxygen was adjusted depending on the respiratory rate. Additionally, mice were kept on a heated animal bed during measurement to avoid a decrease of the body temperature. An eye ointment was administered to prevent the eyes from drying out. The brain was situated at a position inside the scanner with minimal intensity inhomogeneity artifacts and the scanned volume was centered to their brain.

#### MRI-scanning (post-mortem)

Mice were euthanized and quickly transferred (less than 5 min) to the MRI scanner and a 3D T2-weighted turbo-spin echo (TSE) sequence (96 transversal slices, 100 μm thickness, no gap, matrix of 512 × 512 pixel interpolated by the scanner to 1024 × 1024 pixel, FoV = 25 mm, spatial resolution 24 μm × 24 μm × 100 μm, voxel volume 0.058 nl, TR = 2500 ms, TE = 55 ms) was used for the brain scan. The total scan time of the *post-mortem* sequence was 08:38 h. The brain was situated at a position inside the scanner with minimal intensity inhomogeneity artifacts and the scanned volume was centered to their brain.

#### MRI-scanning (ex situ)

Brains were removed by an experienced veterinarian subsequently to the *post-mortem* scan and conserved in 4% formaldehyde. The above described *in vivo* and *post-mortem* sequences were used in direct succession during an *ex situ* scanning session.

### ANATOMICAL RECONSTRUCTION AND LINEAR SHRINKAGE FACTOR

In order to analyze whether the higher resolution of the *post-mortem* MRI scans revealed a more detailed cytoarchitecture, brain areas visible were determined and mapped by the use of an atlas of the mouse brain with stereotaxic coordinates (Paxinos and Franklin, 2001). For visualization pseudo-3D images of the brains were generated, using the tool “maximum intensity projection” implemented in Neurolucida.

For anatomical 3D mapping and reconstruction Neurolucida 10 (MBF Biosciences, USA) was used. Image sequences were loaded into ImageJ 1.44p (NIH, USA) and exported as a sequence of tiff-files. The image sequence of tiff-files was loaded into Neurolucida. The mean thickness of the cortical layer was determined at 12 different positions per brain that were randomly selected. The linear shrinkage factor (Jinno et al., 1999) was determined as change (in percent) as compared to the mean thickness of the cortical layer determined under *in vivo* conditions. For determining whether the thicknesses of the cortex were affected by the treatment, one-way ANOVA, followed by a Tukey’s multiple comparison test was performed using Prism 6.0 (GraphPad Inc., USA).

### PREPROCESSING AND AUTOMATIC SEGMENTATION

Mice brain were segmented into gray matter, white matter and cerebrospinal fluid using SPMMouse (Sawiak et al., 2009), based on the unified segmentation algorithm (Ashburner and Friston, 2005). A proper segmentation for the *in vivo* scans was achieved only if an intensity inhomogeneity correction (IIC) was applied, achieved using a non-parametric method (Sled et al., 1998). *Post-mortem* scans did not need this preparatory processing. Additionally to these segmentations with minimum number of processing steps, a second trial with an increased number of processing steps was carried out. In order to segment without non-brain tissue contributions brain masks were generated as outlined in Section “Sequence Evaluation” and segmentation was applied to the extracted brains. Volume measurements were carried out using the SPMMouse functionality, were volumes are computed by counting the non-zero voxels and multiplying with the voxel volume. The same procedure was applied to the *ex situ* brains.

#### Sequence evaluation

SNR of the entire brain including gray matter, white matter, and cerebrospinal fluid was computed for all unprocessed scans. Therefore, a brain mask was generated for each of the scans. In order to achieve a proper brain mask for the *in vivo* scans three processing steps were necessary. An IIC was carried out to minimize artifacts. Subsequently brains were extracted by hand. The hand extracted brains were again extracted using PCNN3D to achieve a high precise brain mask. In case of the *post-mortem* scans application of PCNN3D sufficed to achieve a highly exact brain mask. The resulting brain mask was applied to the corresponding unprocessed scan and the extracted brain was used for SNR calculations. SNR is defined as signal value *S* over the noise standard deviation σ_noise_

$$SNR=S/\sigma_{noise},$$

where σ_noise_ was extracted from the empty volume included in the borders of the scan. The average SNR (SNR_avg_), and its standard deviation (SNR_σ_), was computed by fitting a Gaussian distribution to the histogram of all pixel SNR values.

Segmentation results for white and gray matter were used as masks to compute the average gray *S*_gray,avg_ and white *S*_white,avg_ matter contrast, as well as their standard deviations (σ_gray_ and σ_white_) in the unprocessed scans. Those were used to compute the average contrast-to-noise ratio (CNR) between gray and white matter and its standard deviation

$$CNR_{avg}=|S_{gray,avg}-S_{white,avg}|/\sigma_{noise}\,\;and\,\;$$

$${CNR}_{\sigma }=\left(\sigma_{gray}+\sigma_{white}\right)/\sigma_{noise}.$$

#### Visualization

ImageJ 1.44p (NIH, USA) was used to visualize particular slices in the gray matter segmentation and the entire gray matter segmentation as a volume image.

## RESULTS

### Description of Structural Datasets

**FIGURE 1:**
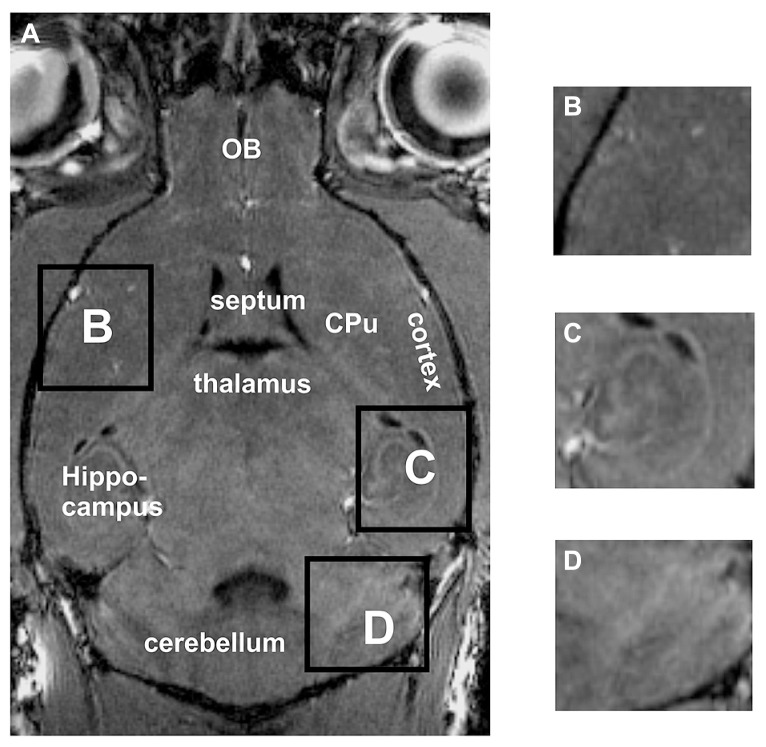
**By using *in vivo* tissue, the brain and the surrounding tissue are clearly visible (A).** However, the boundaries between the cortex and the CPu are difficult to detect **(A**,**B)**. Neither the hippocampal fields CA1, CA3 nor the dentate gyrus can clearly be distinguished **(C)**. Within the cerebellum **(D)** the different layers cannot be differentiated. Inserts **B,C,D** represent magnifications of areas shown in **A**. CPu, caudate putamen; OB, olfactory bulb.

Standard *in vivo* MRI at 7 Tesla allowed to visualize the murine brain and to distinguish major brain regions, e.g., cortex, cerebellum, hippocampus or olfactory bulbs (OB; **Figure 1A**). The cortex and the caudate putamen (CPu) could be well distinguished, but the boundaries between both structures could not be defined exactly (**Figures 1A,B**). Similarly, substructures could not be demonstrated convincingly, e.g., different hippocampal areas (**Figure 1C**) or different layers of the cerebellum (**Figure 1D**).

**FIGURE 2:**
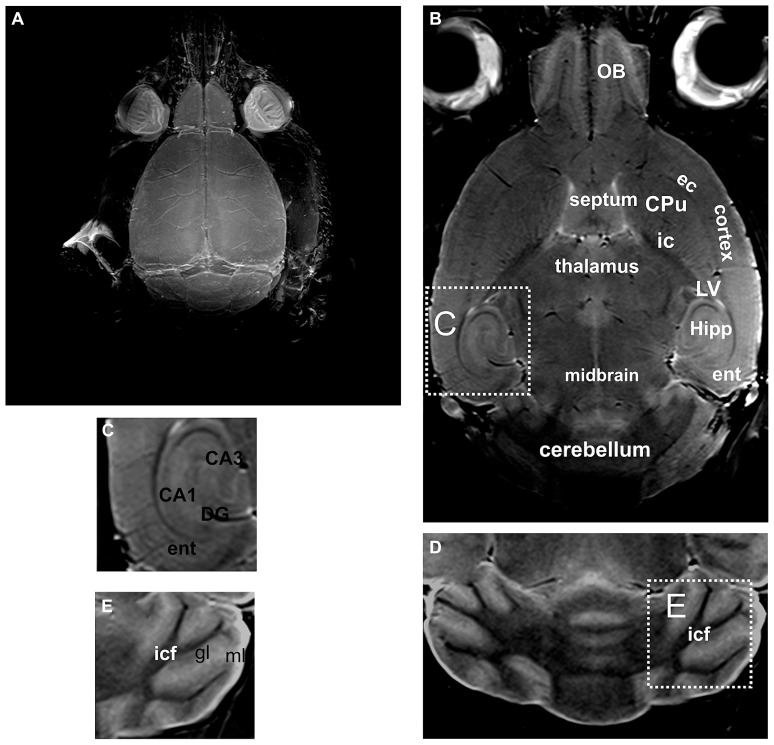
**By using unfixed *post-mortem* tissue, the brain and the blood vessels located on the brain surface are clearly visible in an intensity projection of the 3D scan (A).** A representative slice through a 3D volume of un-fixed *post-mortem* head of a mouse is shown in **(B)**. A higher magnification of the hippocampal formation is shown in **(C)**. Likewise in the cerebellum **(D)** the white matter tracts and the gray matter can be distinguished. However, the Purkinje cell layer, which is composed of a single layer of relatively large neurons, cannot be detected (a higher magnification of this area is shown in **E)**. CA1, CA3, hippocampal area; CPu, caudate putamen; DG, dentate gyrus; ec, external capsule; ent, entorhinal cortex; gl, granular layer of the cerebellum; Hipp, hippocampus; ic, internal capsule; icf, intercrural fibers; LV, lateral ventricle; ml, molecular layer of the cerebellum; OB, olfactory bulb.

*Post-mortem* MRI at 7 Tesla allows visualization of the murine brain in greater detail. Thus, using maximum intensity projection of the entire scan, the brain surface along with the blood vessels was clearly visible (**Figure 2A**). Without use of any fixation or contrast enhancing agents, not only the brain could be distinguished from the surrounding tissue, but also substructures could be differentiated (**Figures 2B,C**). Thus, not only cortical areas could be distinguished from, e.g., the CPu, but also the external capsule could be recognized, as well as different parts of the olfactory bulb, corresponding to different layers of the olfactory bulb (**Figure 2B**). For the cerebellum the white matter tracts and the granular and molecular layer could be distinguished (**Figures 2D,E**) but not the cerebellar Purkinje-cell layer.

**FIGURE 3:**
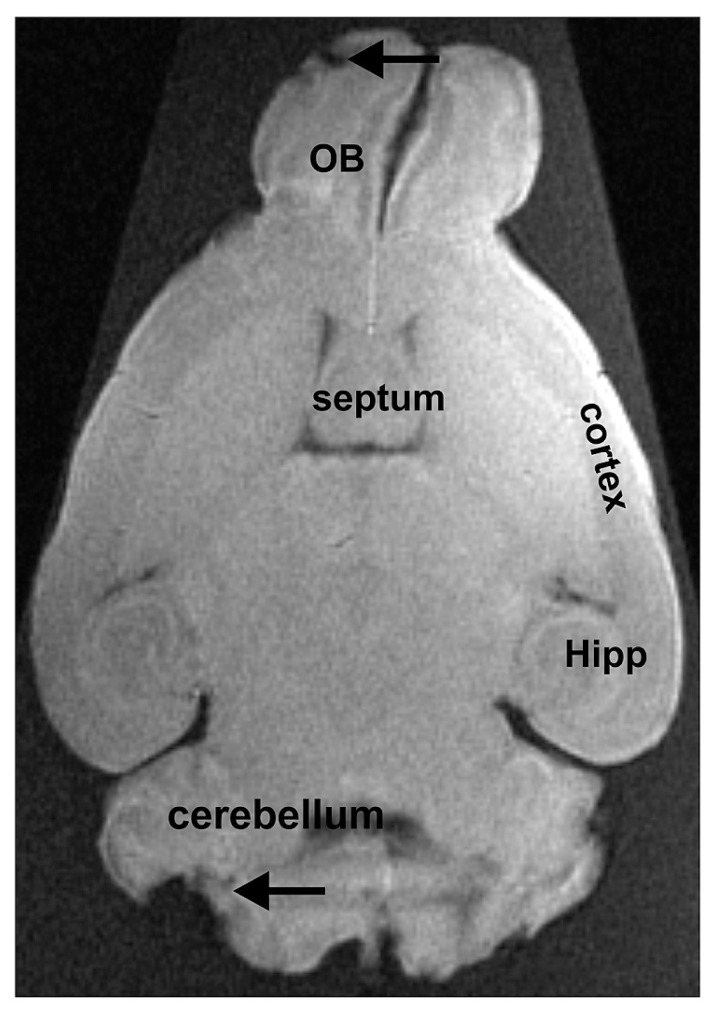
**Extracted brain tissue that was fixed for several days.** The olfactory bulbs (OB), the cortex, the hippocampus (Hipp), and the cerebellum can easily be distinguished from adjacent brain regions. Due to the removal of the brain from the surrounding tissue, some damage can be noted, as e.g., in the cerebellum or the OB (indicated by arrows).

Concerning the fixed extracted brains, the structure was less well preserved (**Figure 3**). This confirms that fixation produces loss in quality (see Introduction section). In addition, brains were in an advanced state of decomposition due to the long delay between euthanasia and extraction. This resulted in partial tissue damage during the extraction procedure. In our approach, the brains were deformed by the flask used for their storage (see **Figure 3**). This latter problem could be overcome by using a different mode of storage.

**FIGURE 4:**
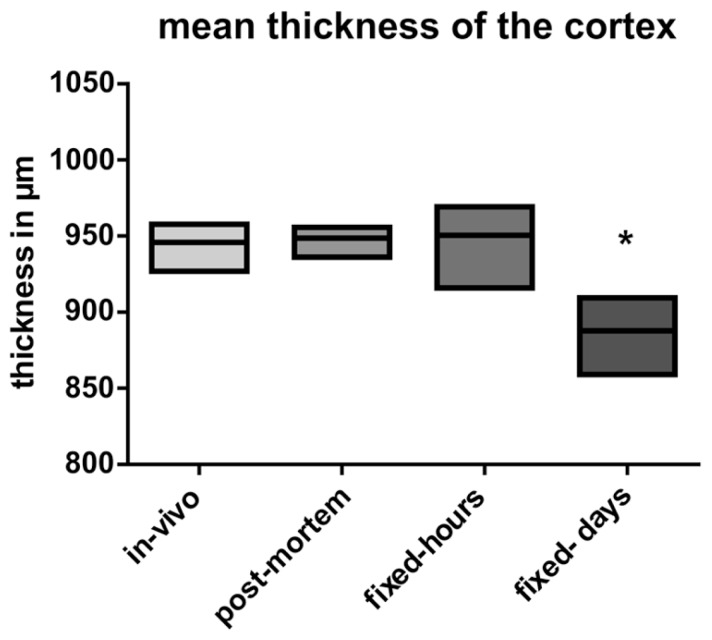
**Analysis of the mean thickness of the cortex.** Data are presented as mean ± min to max (*p ≤ 0.05; ANOVA; Tukey’s *post hoc* test). *In vivo*: data from the *in vivo* scanning procedure; *post-mortem*: data obtained by using *post-mortem* brains; fixed-hours: data obtained by using extracted brains that were fixed for some hours; fixed-days: data obtained from extracted brains that were fixed for several days.

No statistical significant shrinkage for the *post-mortem* brains was observed as compared to the *in vivo* brains (**Figure 4**). Short fixation did also not result in statistical significant shrinkage, but induced a larger variance (**Figure 4**). In contrast, long-term fixation was found to induce a significant shrinkage of the cortical thickness (**Figure 4**).

### PREPROCESSING AND AUTOMATIC SEGMENTATION

**FIGURE 5:**
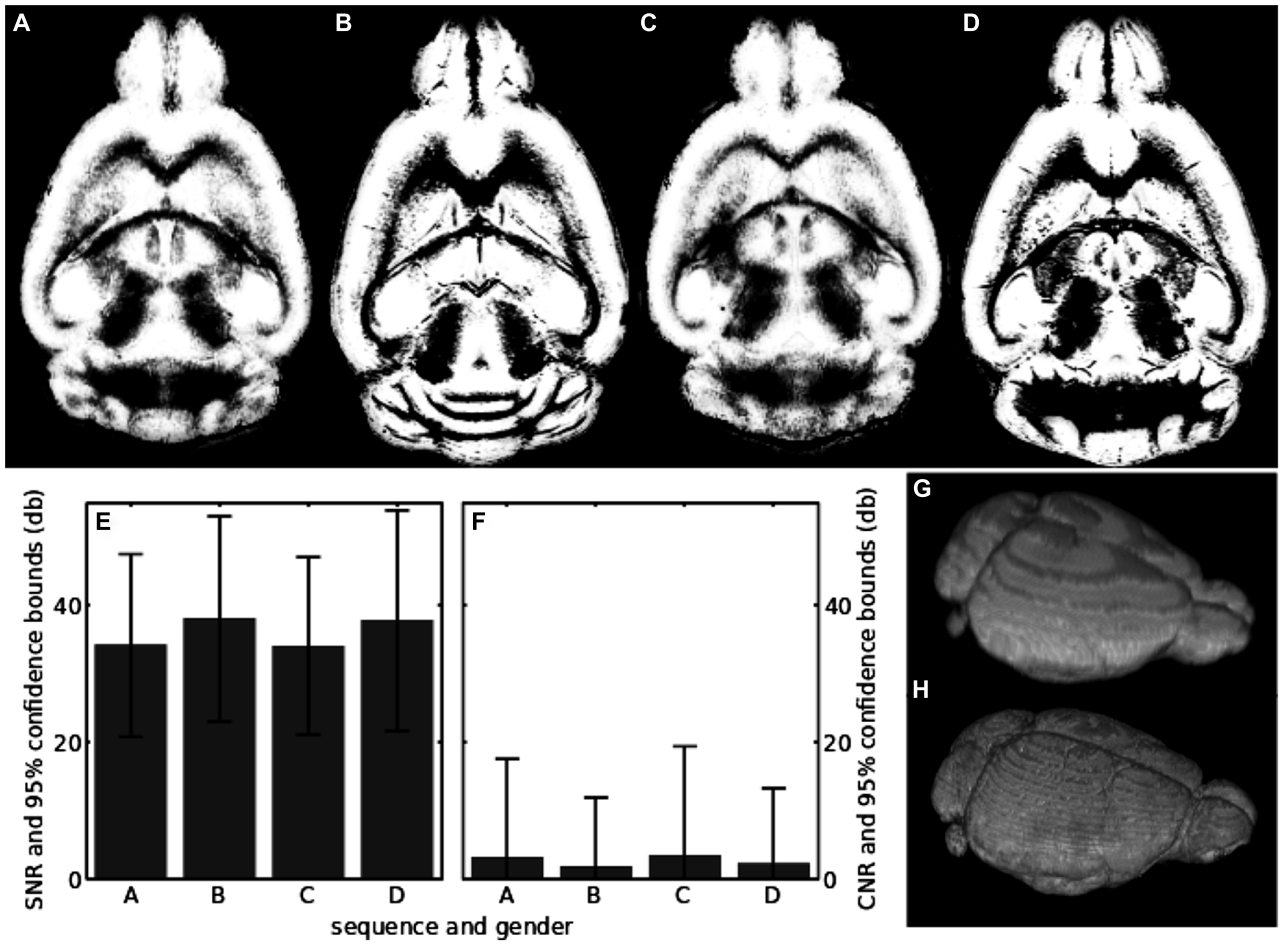
**(A)** Center slice in the segmentation of the *in vivo* sequence for the female mouse; **(B)** same as in **A**, but for the *post-mortem* scan; **(C)** segmentation of the *in vivo* sequence for the male mouse; **(D)** the same as in C, but for the *post-mortem* scan; **(E)** SNR of raw unsegmented *in vivo* and *post-mortem* scans. The labels on the horizontal axis show which sequence and mouse has been analyzed and correspond to the upper row of the figure; **(F)** the same as in **E**, but for the CNR; **(G)** volume visualization of the *in vivo* segmentation result shown in **A**; **(H)** volume visualization of the *post-mortem* segmentation result shown in **B**.

An IIC was necessary to achieve a good segmentation of the *in vivo* scans, which included all brain regions (**Figures 5A,C,G**). Parts of the cerebellum where missing in the segmentation if IIC was not applied. The *post-mortem* scans did not need any preprocessing to achieve an excellent segmentation (**Figures 5B,D,H**). *In vivo* and *post-mortem* segmentations both contained non-brain tissue in the gray matter segmentation, visible in **Figures 5A–D** as white stripes encompassing the outer limit of the gray matter. Brain extraction preceding segmentation decreased the amount of non-brain tissue in the segmentation. Applying IIC or modifying SPMMouse parameters also changed the tissue volumes. However, volumes of *in vivo* and *post-mortem* scans did not converge against identical values regardless of the fine tuning procedures. Therefore, the minimum number of processing steps preceding segmentation and standard SPMMouse parameters were chosen.

This was done to minimize errors due to the processing and to achieve maximum comparability to literature values. In case of *in vivo* scans, the gray matter volumes for the female and male mouse differed by less than 2% (**Table 1**), while the *post-mortem* scans differed by less than 4% (**Table 1**). The maximum difference between *in vivo* and *post-mortem* scan gray matter volume was 8% (see **Table 1**).

**Table 1:** Quantitative results from post-processing.

	Female *in vivo*	Female *post-mortem*	Male *in vivo*	Male *post-mortem*
*V* (ml)	0.3004	0.2779	0.2954	0.288
SNR_avg_	2569.2 (34.1 db)	6253.7 (38 db)	2500.3 (34 db)	5942 (37.7 db)
SNR_σ_	11 (10.4 db)	15.8 (12 db)	10 (10 db)	20.7 (13.2 db)
CNR_avg_	2.1 (3.1 db)	1.5 (1.8 db)	2.2 (3.4 db)	1.7 (2.2 db)
CNR_σ_	14.2 (11.5 db)	5.2 (7.2 db)	20 (13 db)	6.5 (8.1 db)

*
Columns show the results for a particular scan. In the second row, the gray matter volume is shown in milliliters. The third row shows the average SNR and the fourth row the corresponding standard deviation. Average CNR is shown in row five and the corresponding standard deviation in row six.*

The SNRs of all scans together with their standard deviations are shown in **Table 1** and depicted in **Figure 5E**. *In vivo* scans exhibited an SNR_avg_ > 34 db and *post-mortem* scans had an SNR_avg_ > 37 db. All scans had a lower limit of the SNR_avg_ > 20 db if the 95% confidence bound was taken into account. Thus, the brain signal of the image was at least 100 times larger than the noise. The corresponding results for the CNR of all scans and its standard deviation are also shown in **Table 1** and depicted in **Figure 5F**. *In vivo* scans exhibited a CNR_avg_ > 2.1 db and *post-mortem* scans had a CNR_avg_ > 1.5 db. Thus, gray and white matter contrasts differ on average about 50% in their contrast values. Notably, CNR_avg_ of *in vivo* scans was slightly larger than of *post-mortem* scans. The opposite was found for the standard deviations of the CNRs, which was probably due to a higher effect of intensity inhomogeneities in the *in vivo* scans. CNR_σ_ was larger than CNR_avg_ by at least a factor 6 for the *in vivo* scans, while it was at least a factor of 3 for the *post-mortem* scans.

## DISCUSSION

We demonstrate that the use of non-fixed *post-mortem* tissue is well-suited as a quick and sensitive method for high-resolution MRI. Several technical difficulties found previously at 7 Tesla MRI (Beuf et al., 2006) were dissolved, leading to high-throughput capabilities. Damage due to the removal of the brains and time-consuming tissue processing, possibly leading to severe shrinkage or other fixation artifacts, are avoided. Compared to the *in vivo* situation, artifacts due to respiration and cardiac activity are absent and longer scan times are possible. This allows applying T2-weighted sequences with high spatial resolution and contrast, as compared to fast T1-weighted sequences applied *in vivo*.

The qualitative inspection of *in vivo*, *post-mortem* and *ex situ* scans confirmed the results found in the literature concerning *in vivo* and *ex situ* scans. An identification of substructures in *in vivo* scans was difficult due to the low quality of the scans, but could be achieved at various positions for the cortex. In *ex situ* scans fixation artifacts, deformations and damages affected the scans severely and complicated the reliable identification of brain substructures. These artifacts may occur during the formalin fixation process (Benveniste and Blackband, 2006).

The thickness analysis of the cortical layer indicated that significant shrinkage was found for brains fixated for days, while no shrinkage was found if brains were fixated for hours only or without fixation. Along this line, in a recent MRI study, using human *post-mortem* brain that have been fixed with formalin, it has been shown that fixation resulted after several days in substantial tissue shrinkage and local deformations (Schulz et al., 2011). In histology, the impact of different fixatives on tissue shrinkage is well known (Stickland, 1975) and recently the impact of formaldehyde fixation on distortion of brain tissue has been re-examined in detail (Weisbecker, 2012). Since shrinkage of the tissue was found to have an impact on stereological estimates, a volumetric shrinkage factor has been introduced (Jinno et al., 1999). Paraformaldehyde fixation, sectioning and staining of mouse brain sections, and subsequent embedding can produce a volumetric shrinkage factor of about 0.6 (von Bohlen und Halbach and Unsicker, 2002). Thus, post-processing of the tissue, like the use of contrast enhancing substances on formaldehyde fixated brains for MRI scans, may let to further tissue distortion. Techniques developed for shrinkage correction in histological specimens are unfortunately not applicable for MR imaging analysis. However, algorithms compensating for volumetric shrinkage and tissue distortion due to fixation (Schulz et al., 2011) are not necessary if non-fixated *post-mortem* brains are investigated *in situ*.

Overall, the *post-mortem* scans allowed for a more distinct identification of substructures than the *in vivo* scans, and exhibited minimal influence of artifacts. This was also confirmed for the entire gray matter in the *post-mortem* scan using IIC and SPMMouse brain segmentation techniques. The maximum difference between *in vivo* and *post-mortem* scan gray matter volume was 8%, which confirms the result for cortical thickness depicted in **Figure 4**. Only one *ex situ* brain dataset allowed for an automatized segmentation. This was due to alterations in the brain caused by fixation, extraction and/or deformation during storage. Since the *ex situ* brains have to be investigated in carriers, a positioning of surface MRI-coils near the brain is hampered, too. This might also decrease MRI-signal intensity, which might well result in problems in segmentation (Ashburner and Friston, 2005).

The contrast-to-noise ratio (CNR) is highly important for the differentiation between tissues in MRI scans. SNR of the scans is excellent at 7T and shows relevant improvement if compared to MRI at lower field strengths (Natt et al., 2002). However, the CNR is rather small and changes considerably between measurements. On average gray and white matter contrasts differ by 50% in their contrast values in the *in vivo* and *post-mortem* scans. Since the quality of the automatic segmentation will depend on CNR, optimization of MRI sequences at 7 Tesla should aim on improving the CNR. This could be achieved in the post-processing using an IIC. Further optimization of sequence parameters might result in improved CNR (DiFrancesco et al., 2008) and minimized intensity inhomogeneities (Tannus and Garwood, 1997).

Cleary and colleagues described that the usage of contrast enhancing agent gadolinium on fixated brains in a 9.4 Tesla scanner allowed for visualization of the Purkinje cell layer of the cerebellum (Cleary et al., 2011). In our approach, the resolution was not high enough to distinguish single Purkinje cells or other single neurons; a problem that might be overcome by using a 9.4 T scanner. The use of the *post-mortem* MRI technique is, however, much faster and may therefore be a valuable method for anatomical phenotyping of transgenic mice. In addition, it enables an increase in the throughput for MRI phenotyping of large numbers of rodents, allowing to determine very quickly small structural alterations in the murine brain at a reasonably high resolution even with 7 Tesla field strength. Furthermore, an increase in scanning times will no longer represent a limiting factor for obtaining higher resolutions.

## Conflict of Interest Statement

The authors declare that the research was conducted in the absence of any commercial or financial relationships that could be construed as a potential conflict of interest.
